# Lipodermatosclerosis-like clinical presentation with PXE-like histopathology in a patient with newly diagnosed vascular Ehlers-Danlos syndrome

**DOI:** 10.1016/j.jdcr.2026.04.039

**Published:** 2026-04-29

**Authors:** Olivia Graham, Kavish Singh, Peter Chow, Urmi Khanna

**Affiliations:** a Division of Dermatology, University of Kansas Medical Center, Kansas City, Kansas; b Division of Rheumatology, Dartmouth Hitchcock Medical Center and Geisel School of Medicine at Dartmouth, Lebanon, New Hampshire; c Department of Dermatology, Dartmouth Hitchcock Medical Center and Geisel School of Medicine at Dartmouth, Lebanon, New Hampshire

**Keywords:** lipodermatosclerosis, pseudoxanthoma elasticum, vascular Ehlers-Danlos syndrome

## Introduction

Vascular Ehlers-Danlos syndrome (vEDS) is a rare heritable connective tissue disorder caused by a heterozygous pathogenic variant in COL3A1, leading to defective synthesis and secretion of type III collagen.^1^ This defect can present with characteristics including thin, translucent skin, easy bruising, joint hypermobility, acrogeria, and spontaneous vascular and visceral rupture.^2^

Pseudoxanthoma Elasticum (PXE) is another distinct heritable connective tissue disorder, with some overlapping clinical features, particularly in vascular involvement. PXE is caused by autosomal recessive mutations in the ABCC6 gene, which leads to defective mineralization and fragmentation of elastic fibers, eventually resulting in vascular calcification.^3^

We describe the distinctive case of a 66-year-old woman who developed new onset skin sclerosis clinically mimicking lipodermatosclerosis (LDS) occurring in the context of a newly established diagnosis of vEDS with histopathologic evaluation demonstrating PXE-like alterations.

## Case report

A 66-year-old female presented with tightness, and swelling of both legs, associated with progressive purplish discoloration, coolness, and some numbness. She reported a 40-pound weight gain after discontinuation of tirzepatide. Her past medical history included type 2 diabetes mellitus, hypertension, hyperlipidemia, sleep apnea, iron deficiency anemia, arthritis, diverticulitis, and gastroesophageal reflux disease. Her surgical history was significant for spontaneous bowel perforation requiring colostomy with failed takedown due to severe rectal friability. Her clinical course was further complicated by recurrent incisional ventral hernia. Family history was notable for multiple relatives carrying a COL3A1 mutation and/or history of perforated diverticulosis.

**Fig 1:**
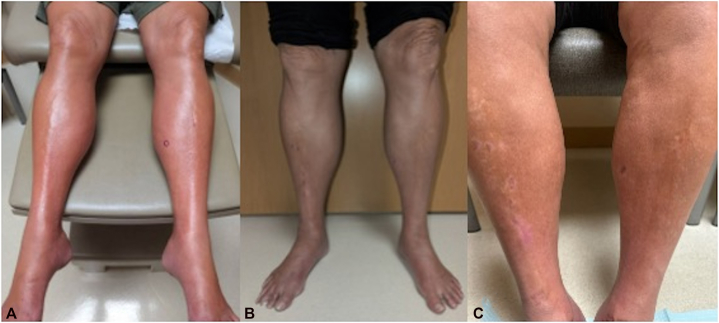
Clinical presentation. Localized sclerosis of bilateral lower extremities at initial presentation **(A)**. Bilateral lower extremities 2 months after initiation of potent topical steroids, and pentoxifylline **(B)**. Close up view of the lower extremities 6 months after initiation of treatment **(C)**.

**Fig 2:**
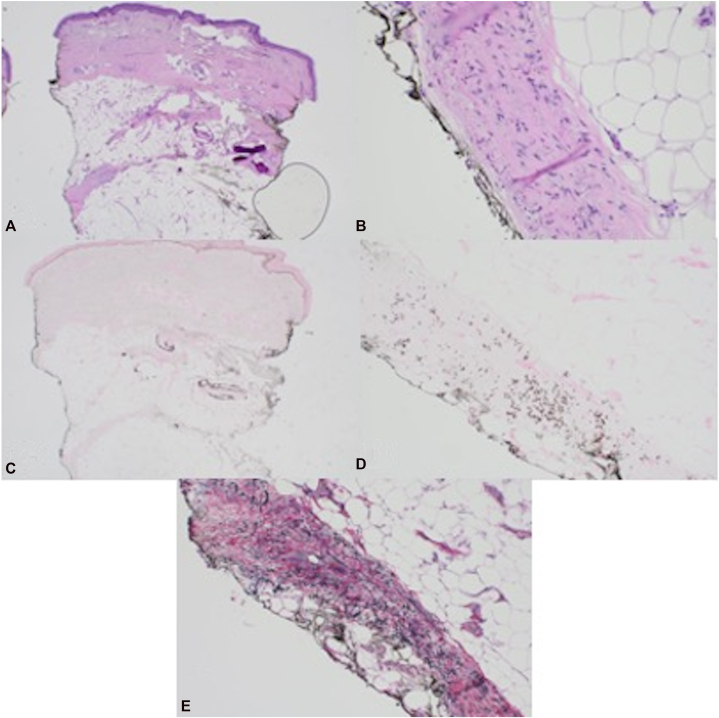
Histologic findings on punch biopsy.Hemolysin and Eosin staining **(A** and **B),** Von Kossa calcium staining **(C** and **D)**, and Verhoeff-Van Gieson elastic staining **(E)** together highlight the calcified elastic fibers in the septum of the subcutis.

Physical examination revealed localized sclerosis of bilateral lower extremities, extending from the dorsum of the feet to the knees. The skin appeared indurated, shiny, and exhibited an “inverted champagne bottle” appearance (Fig 1, *A*); the remainder of the cutaneous exam was unremarkable. Laboratory evaluations showed antinuclear antibody and extractable nuclear antigen panels were negative. A punch biopsy from the left lower extremity demonstrated PXE-like changes and calcification (Fig 2). Prior to dermatologic evaluation, a doppler ultrasound demonstrated no venous insufficiency. During the subsequent workup, computed tomography angiography demonstrated moderate stenosis of the superior mesenteric artery (SMA) without symptoms of mesenteric ischemia on further workup.

With the patient’s significant family history, unsuccessful attempt at colostomy takedown, and PXE-like changes on skin biopsy, genetic testing was pursued and identified a COL3A1 pathogenic mutation confirming the diagnosis of vEDS. The patient was treated with potent topical steroids and pentoxifylline, and subsequently reported decreased skin tightness, reduced swelling, and improvement of nighttime discomfort (Fig 1, *B* and *C*).

## Discussion

The patient’s strong family history of vEDS, prior spontaneous bowel perforation, recurrent hernia and a skin biopsy performed to evaluate new onset leg sclerosis demonstrating PXE-like changes prompted referral for genetic testing for Ehlers Danlos syndrome, which confirmed a COL3A1 mutation.^4^^,^^5^ She was referred for close multidisciplinary follow-up, including genetics, vascular surgery for surveillance of SMA stenosis, colon and rectal surgery for gastrointestinal monitoring, rheumatology, dermatology for treatment of skin thickening, and internal medicine for optimization of diabetes, hyperlipidemia, and weight management. She also follows with outside ophthalmology for her narrow angle glaucoma.

We present this case due to the interesting clinical and histological aspects. The new onset skin sclerosis, clinically resembling LDS but occurring in the absence of venous insufficiency and demonstrating PXE-like elastic fiber changes on a representative skin biopsy, led the pathologist to suspect an underlying connective tissue abnormality.^6^, ^7^, ^8^ We believe that in this patient the LDS-like findings could be due to secondary fibrotic changes related to venous stasis or vascular fragility characteristic of vEDS. Chronic vascular injury or inflammation could promote elastic fiber degeneration, resulting in PXE-like histopathology. Previous research has shown cases of PXE-like elastic fiber changes in a variety of conditions, including inflammatory skin diseases and noninflammatory elastic tissue disorders, without systemic manifestations of PXE. PXE-fibers have been reported in patients with inflammatory dermatoses such as granuloma annulare, lichen sclerosis, morphea profunda, lipodermatosclerosis, erythema nodosum, septal panniculitis, fibrosing dermatitis and in association with basal cell carcinoma tissue.^9^ Noninflammatory PXE-like conditions have also been described, including papillary dermal elastolysis and focal dermal elastosis. Papillary dermal elastosis is characterized by complete loss or decreased elastic fibers in papillary dermis, whereas focal dermal elastosis shows accumulation of normal elastic fibers in the reticular dermis.^10^

This case raises the possibility that vascular fragility associated with vEDS may predispose patients to venous insufficiency, fibrosis, and elastic tissue degeneration, resembling the features of LDS clinically and consequently showing PXE-like changes histopathologically.

In summary, we present an atypical clinicopathologic case of vEDS demonstrating LDS-like clinical findings with PXE-like histopathology. Further studies are needed to determine whether this overlap represents a mechanistic association or a coincidental finding. Clinicians should be aware of this potential presentation in patients with vEDS who present with lower extremity fibrosis or atypical histopathologic features.

## Conflicts of interest

None disclosed.

## References

[1] Shalhub S., Byers P.H., Hicks K.L. (2020). A multi-institutional experience in vascular Ehlers-Danlos syndrome diagnosis. J Vasc Surg.

[2] Byers P.H., Adam M.P., Feldman J., Mirzaa G.M. (1993).

[3] Pfau K., Lengyel I., Ossewaarde-van Norel J. (2024). Pseudoxanthoma elasticum - genetics, pathophysiology, and clinical presentation. Prog Retin Eye Res.

[4] Doolan B.J., Lavallee M., Hausser I. (2023). Dermatologic manifestations and diagnostic assessments of the Ehlers-Danlos syndromes: a clinical review. J Am Acad Dermatol.

[5] Ishikawa S., Hayashi S., Sairenchi T. (2023). Clinical features and morphology of collagen fibrils in patients with vascular Ehlers-Danlos based on electron microscopy. Front Genet.

[6] Geist R.S., Crane J.S. (2023). Lipodermatosclerosis. StatPearls [Internet]. Treasure Island (FL).

[7] Alsararatee H.H. (2025). Lipodermatosclerosis: from pathophysiology to treatment. Br J Nurs.

[8] Walsh S.N., Santa Cruz D.J. (2010). Lipodermatosclerosis: a clinicopathological study of 25 cases. J Am Acad Dermatol.

[9] Bowen A.R., Gotting C., LeBoit P.E., McCalmont T.H. (2007). Pseudoxanthoma elasticum-like fibers in the inflamed skin of patients without pseudoxanthoma elasticum. J Cutan Pathol.

[10] Aljoudi S.B., Abduljabbar M.H., Hariri J.O. (2019). A case series of pseudoxanthoma elasticum-like disorders. Indian J Dermatol.

